# Supplementary material for: Lipotype acquisition during neural development is not recapitulated in stem cell–derived neurons
Source: Life Sci Alliance. 2024 Feb 28;7(5):e202402622. doi: 10.26508/lsa.202402622 (PMC10902711; doi:10.26508/lsa.202402622)
Supplement: Supplementary file 2 [file LSA-2024-02622_TableS1.docx]

##### **Table S1.** Technical details related to identification and quantification of lipids of various classes.

| # | Lipid class | Abbreviation | Internal standard | Spike amount (pmol) | Extract phase (C/M) | Ion mode | Adduct | MS m/z scan range |
| --- | --- | --- | --- | --- | --- | --- | --- | --- |
| 1 | Acylcarnitine | ACar | IS ACar 16:0(+^2^H_3_) | 38 | 10:1 | +ve | +H^+^ | 345-605 |
| 2 | Cholesterol ester | CE | IS CE 10:0 | 531 | 10:1 | +ve | +NH4^+^ | 470-1400 |
| 3 | Ceramide | Cer | IS Cer 18:1;2/17:0;1 | 103.5 | 10:1 | -ve | +HCOO^-^ | 500-1400 |
| 4 | Cardiolipin | CL | IS CL 14:1/14:1/14:1/15:1 | 69.95 | 2:1 | -ve | -2H^+^ | 530-1080 |
| 5 | Diacylglycerol | DAG | IS DAG(+^2^H_5_) 17:0/17:0 | 109.8 | 10:1 | -ve | +HCOO^-^ | 500-1400 |
| 6 | Monosialodihexosy-ganglioside | GM3 | IS PI 15:0/18:1(+^2^H_7_) | 51.85 | 2:1 | -ve | -H^+^ | 1000-1400 |
| 7 | Hexosylceramide | HexCer | IS HexCer 18:1;2/12:0 | 135 | 10:1 | -ve | +HCOO^-^ | 500-1400 |
| 8 | Lysophosphatidic acid | LPA | IS LPA O-16:0 | 111 | 2:1 | -ve | -H^+^ | 360-675 |
| 9 | Lysophosphatidyl-choline | LPC | IS LPC 16:0(+^2^H_3_) | 28 | 10:1 | -ve | +HCOO^-^ | 350-600 |
| 10 | Lysophosphatidyl-  ethanolamine | LPE | IS LPE O-16:0 | 200.5 | 10:1 | -ve | -H^+^ | 350-600 |
| 11 | Ether lysophosphatidyl-  ethanolamine | LPE O- | IS LPE O-16:0 | 200.5 | 10:1 | -ve | -H^+^ | 350-600 |
| 12 | Lysophosphatidyl-  inositol | LPI | IS LPI 17:1 | 38.7 | 2:1 | -ve | -H^+^ | 530-1080 |
| 13 | Lysophosphatidyl-serine | LPS | IS LPS 17:1 | 12.9 | 2:1 | -ve | -H^+^ | 360-675 |
| 14 | Monolysocardiolipin | MLCL | IS CL 14:1/14:1/14:1/15:1 | 69.95 | 2:1 | -ve | -2H^+^ | 530-1080 |
| 15 | Phosphatidic acid | PA | IS PA 15:0/18:1(+^2^H_7_) | 93.95 | 2:1 | -ve | -H^+^ | 530-1080 |
| 16 | Phosphatidylcholine | PC | IS PC 16:0(+^2^H_3_)/16:0(+^2^H_3_) | 129.3 | 10:1 | -ve | +HCOO^-^ | 500-1400 |
| 17 | Ether phosphatidyl-  choline | PC O- | IS PC 16:0(+^2^H_3_)/16:0(+^2^H_3_) | 129.3 | 10:1 | -ve | +HCOO^-^ | 500-1400 |
| 18 | Phosphatidylethanol-amine | PE | IS PE 15:0/18:1(+^2^H_7_) | 152.85 | 10:1 | -ve | -H^+^ | 500-1400 |
| 19 | Ether phosphatidyl- ethanolamine | PE O- | IS PE 15:0/18:1(+^2^H_7_) | 152.85 | 10:1 | -ve | -H^+^ | 500-1400 |
| 20 | Phosphatidylglycerol | PG | IS PG 15:0/18:1(+^2^H_7_) | 51.2 | 2:1 | -ve | -H^+^ | 530-1080 |
| 21 | Phosphatidylinositol | PI | IS PI 15:0/18:1(+^2^H_7_) | 51.85 | 2:1 | -ve | -H^+^ | 530-1080 |
| 22 | Phosphatidylserine | PS | IS PS 15:0/18:1(+^2^H_7_) | 68 | 2:1 | -ve | -H^+^ | 530-1080 |
| 23 | Sphingomyelin | SM | IS SM 18:1;2/18:1(+^2^H_9_) | 113 | 10:1 | +ve | +H^+^ | 470-1400 |
| 24 | Cholesterol | ST | IS Cholesterol(+^2^H_7_) | 3000 | 10:1 | +ve | +H^+^  -H2O | msx 369.3516 & 376.3955 |
| 25 | Sulfatide | SHexCer | IS SHexCer 18:1;2/12:0 | 22.5 | 2:1 | -ve | -H^+^ | 530-1080 |
| 26 | Triacylglycerol | TAG | IS TAG(+^2^H_5_) 17:0/17:1/17:0 | 60 | 10:1 | +ve | +NH4^+^ | 470-1400 |
